# Supplementary material for: Correction: Mutant TDP-43 Deregulates AMPK Activation by PP2A in ALS Models
Source: PLoS One. 2014 Apr 16;9(4):e95549. doi: 10.1371/journal.pone.0095549 (PMC3989332; doi:10.1371/journal.pone.0095549)
Supplement: File S2 — Republished, corrected article. [file pone.0095549.s002.pdf]

Mutant TDP-43 Deregulates AMPK Activation by PP2A in ALS Models

Nirma D. Perera[1,2], Rebecca K. Sheean[1,2], John W. Scott[3], Bruce E. Kemp[3], Malcolm K. Horne[1,2], Bradley J. Turner[1,2]*

1 Florey Institute of Neuroscience and Mental Health, University of Melbourne, Parkville, Melbourne, Victoria, Australia, **2** Centre for Neuroscience, University of Melbourne, Parkville, Melbourne, Victoria, Australia, **3** St Vincent's Institute and Department of Medicine, University of Melbourne, Parkville, Melbourne, Australia

Abstract

Bioenergetic abnormalities and metabolic dysfunction occur in amyotrophic lateral sclerosis (ALS) patients and genetic mouse models. However, whether metabolic dysfunction occurs early in ALS pathophysiology linked to different ALS genes remains unclear. Here, we investigated AMP-activated protein kinase (AMPK) activation, which is a key enzyme induced by energy depletion and metabolic stress, in neuronal cells and mouse models expressing mutant superoxide dismutase 1 (SOD1) or TAR DNA binding protein 43 (TDP-43) linked to ALS. AMPK phosphorylation was sharply increased in spinal cords of transgenic SOD1^{G93A} mice at disease onset and accumulated in cytoplasmic granules in motor neurons, but not in pre-symptomatic mice. AMPK phosphorylation also occurred in peripheral tissues, liver and kidney, in SOD1^{G93A} mice at disease onset, demonstrating that AMPK activation occurs late and is not restricted to motor neurons. Conversely, AMPK activity was drastically diminished in spinal cords and brains of presymptomatic and symptomatic transgenic TDP-43^{A315T} mice and motor neuronal cells expressing different TDP-43 mutants. We show that mutant TDP-43 induction of the AMPK phosphatase, protein phosphatase 2A (PP2A), is associated with AMPK inactivation in these ALS models. Furthermore, PP2A inhibition by okadaic acid reversed AMPK inactivation by mutant TDP-43 in neuronal cells. Our results suggest that mutant SOD1 and TDP-43 exert contrasting effects on AMPK activation which may reflect key differences in energy metabolism and neurodegeneration in spinal cords of SOD1^{G93A} and TDP-43^{A315T} mice. While AMPK activation in motor neurons correlates with progression in mutant SOD1-mediated disease, AMPK inactivation mediated by PP2A is associated with mutant TDP-43-linked ALS.

Citation: Perera ND, Sheean RK, Scott JW, Kemp BE, Horne MK, et al. (2014) Mutant TDP-43 Deregulates AMPK Activation by PP2A in ALS Models. PLoS ONE 9(3): e90449. doi:10.1371/journal.pone.0090449

Editor: Stefano L Sensi, University G. D'Annunzio, Italy

Received November 13, 2013; **Accepted** February 1, 2014; **Published** March 4, 2014

Copyright: © 2014 Perera et al. This is an open-access article distributed under the terms of the Creative Commons Attribution License, which permits unrestricted use, distribution, and reproduction in any medium, provided the original author and source are credited.

Funding: This work was supported by the Australian National Health and Medical Research Council (Project Grant 1008910), MND Research Institute of Australia (Mick Rodger Benalla and Susie Harris Memorial Fund MND Research Grants), Bethlehem Griffiths Research Foundation and Victorian Government through the Operational Infrastructure Scheme. N.P. is supported by an Australian Postgraduate Award Scholarship. The funders had no role in study design, data collection and analysis, decision to publish, or preparation of the manuscript.

Competing Interests: The authors have declared that no competing interests exist.

* E-mail: bradley.turner@florey.edu.au

Introduction

Amyotrophic lateral sclerosis (ALS) is a progressive and fatal paralysing disorder caused by selective degeneration of upper and lower motor neurons [1,2]. The reason for the selective vulnerability of motor neurons to mutations in ubiquitously expressed proteins, such as superoxide dismutase 1 (SOD1) and TAR DNA-binding protein-43 (TDP 43), remains unclear [1,2]. Factors accounting for this selective neuronal susceptibility in ALS may include the unusual high synthetic, energetic and transport demands of large projection motor neurons [3]. This leads to high ATP consumption and mitochondrial metabolism relative to other cells, rendering these neurons susceptible to energetic defects [4]. Thus, defects in mitochondrial function that occur in ALS patients and mouse models [5,6] will affect ATP production, impairing Na^+/K^+-ATPase function and maintenance of resting membrane potential in motor neurons [7].

There is considerable evidence for hypermetabolism and impaired energy homeostasis in ALS patients and genetic mouse models. ALS patients show accelerated loss of muscle mass and fat during disease progression [8,9] and elevated energy expenditure at rest [10,11]. In mutant SOD1^{G86R} and SOD1^{G93A} mice, glucose, ATP and fat metabolism are increased in spinal cord and brain at presymptomatic disease, indicative of energy hypermetabolism [12,13]. In symptomatic SOD1^{G93A} mice, there is increased metabolic acidosis, lipolysis and glycogen accumulation in the CNS [14]. Furthermore, a high-fat diet significantly delayed disease onset and increased lifespan in mutant SOD1 mice [12], while caloric restriction accelerated disease course [15,16].

There is also evidence for metabolic dysfunction in ALS mediated by abnormal TDP-43. Postnatal depletion of TDP-43 in mice leads to dramatic loss of body fat followed by rapid death and *Tbc1d1* expression, a gene that mediates leanness and linked to obesity, is downregulated in the absence of TDP-43 [17]. In TDP-43^{A315T} mice, there is progressive weight gain, fat deposition, impaired glucose uptake and increased *Tbc1d1* expression in presymptomatic mice [18]. Together, this suggests that TDP-43 expression level is an important regulator of glucose and energy metabolism, while more importantly, mutant TDP-43 leads to metabolic dysfunction in an opposing manner to mutant SOD1.

Here, we sought to correlate these well established findings of defective energy metabolism in mutant SOD1 and TDP-43 mice

with AMP-activated protein kinase (AMPK) activation status. AMPK is a widely expressed master metabolic and stress sensor which detects mismatches in cell energy supply and demand [19]. It is activated by high AMP:ATP ratio and metabolic stresses that inhibit ATP production or stimulate ATP consumption [19]. AMPK is a heterotrimeric protein consisting of α catalytic and regulatory β and γ subunits. Binding of AMP to the γ subunit stimulates α subunit phosphorylation at the active site threonine-172 by two known upstream AMPK kinases, liver kinase B1 (LKB1) and Ca^{2+}/calmodulin-dependent protein kinase kinase (CaMKK) as reviewed [19]. AMPK inactivation by dephosphorylation has been linked to protein phosphatase 2A (PP2A) [20], PP2C [21], PP1 [22] and recently PPM1E [23]. Activation of AMPK replenishes cellular ATP levels by promoting increased fatty acid oxidation, glucose uptake and autophagy, while reducing protein translation, glycogen and cholesterol synthesis.

Since AMPK is a master regulator of energy metabolism and stress induced pathways, it is likely to be engaged in energetic defects in motor neurons in ALS. We therefore examined AMPK activation in $SOD1^{G93A}$ and $TDP\text{-}43^{A315T}$ mice and neuronal cells expressing different ALS-linked SOD1 or TDP-43 mutants. Here, we show that AMPK activation is increased in spinal cords of $SOD1^{G93A}$ mice consistent with energy hypermetabolism, while AMPK activation is severely reduced in CNS tissues of $TDP\text{-}43^{A315T}$ mice, in accordance with weight gain and fat accumulation characteristic of this model. We also uncover a novel regulation of AMPK activation by mutant TDP-43 via PP2A.

Methods

Ethics statement

All experiments conformed to the Australian National Health and Medical Research Council published code of practice and were approved by the Howard Florey Institute Animal Ethics Committee.

Transgenic mice

Transgenic $SOD1^{G93A}$ mice derived from the B6SJL-TgN(SOD1*G93A)1Gur line were obtained from the Jackson Laboratory (Bar Harbor, ME) and backcrossed onto a pure C57BL/6 background. Non-transgenic littermates of $SOD1^{G93A}$ mice were used as their wild-type (WT) controls. Transgenic $TDP\text{-}43^{A315T}$ mice derived from the B6.Cg-Tg(Prnp-TARDBP*A315T)95Balo/J line (Jackson Laboratory) were maintained on a C57BL/6 background. Non-transgenic littermates of $TDP\text{-}43^{A315T}$ mice were used as their WT controls. Only male mice were analysed in this study due to the substantial sex differences in survival of $TDP\text{-}43^{A315T}$ mice.

Tissue extraction

Animals were killed by lethal injection (100 mg/kg, intraperitoneal, sodium pentobarbitone) at postnatal day (P) 60 or 90 and lumbar spinal cord, whole brain, kidney and liver were dissected out and snap-frozen. Tissues were homogenised in RIPA lysis buffer containing 50 mM Tris-Cl, pH 7.4, 150 mM NaCl, 0.1% SDS, 1% sodium deoxycholate, 1% TX-100, 1% protease inhibitor cocktail (Sigma) and phosphatase inhibitors (50 mM NaF and 10 mM sodium pyruvate) and sonicated at 50% output for 15 sec, stored on ice for 20 min and centrifuged at 15,800 *g* for 20 min at 4°C to collect supernatants. Proteins were quantified using the bicinchoninic acid assay kit (Pierce) using bovine serum albumin standards.

Stable cell line generation

Mouse neuroblastoma x spinal cord (NSC-34) cells were cultured in Dulbecco's Modified Eagle Medium with 10% heat-inactivated fetal bovine serum, 1% penicillin-streptomycin and 1% glutamine (Invitrogen). pEGFP-N1 vector (Clontech) containing human wild-type (WT) or mutant (A4V, G37R, G85R, G93A) SOD1 cDNAs with a C-terminal EGFP tag were generated as previously described [24]. pmCherry-N1 vector (Clontech) containing human WT or mutant (D169G, A315T, Q331K, M337V) TDP-43 cDNAs with a C-terminal mCherry tag were generated by Dr. Adam Walker and Mr. Yi Ma. For stable cell lines, cells were subcultured and transfected in 6-well plates with SOD1 or TDP-43 constructs by selection in 700 µg/ml G418 (Promega) for 2-weeks and limiting dilution was used to generate monoclonal cell lines. Clones were chosen with endogenous expression level of human SOD1 or TDP-43.

Cell lysis

Cells were lysed in buffer containing 20 mM Tris-Cl, pH 7.5, 150 mM NaCl, 1% Triton-X 100, 1% (v/v) protease inhibitor cocktail (Sigma) and phosphatase inhibitors (50 mM NaF and 10 mM sodium pyruvate) for 20 min on ice. Lysates were centrifuged at 14, 000 rpm for 20 min at 4°C to collect supernatants. Proteins were quantified as above for tissue.

AMPK activation assay

Cells were subcultured in 6-well plates (5×10^5 cells/well) or on glass coverslips (Grale Scientific) in 24-well plates (5×10^4 cells/well) and treated with 5-aminoimidazole-4-carboxamide riboside (AICAR) (Sigma) for 2 hr. Cells grown in 6-well plates were lysed for immunoblot analysis and 24-well plates were used for immunocytochemistry.

PP2A inhibition

Cells were subcultured in 6-well plates (5×10^5 cells/well) and treated with 0.1–1 µM okadaic acid (Tocris) for 30 min. Cells were lysed for immunoblot analysis.

Cell viability assay

Cells were subcultured in 96-well plates (1×10^4 cells/well) and treated with AICAR for 2 hr. Cell viability was assessed by reduction of 3-(4,5-dimethylthiazol-2-yl)-2,5-diphenyltetrazolium bromide (MTT) (Sigma). Cells were treated with MTT (0.5 mg/ml) for 1 h at 37°C, medium was aspirated and cells were solubilised in DMSO for absorbance measurements at 530 nm.

Immunoblotting

Proteins (50 µg tissue and 20 µg cells) were electrophoresed through 12.5% SDS polyacrylamide gels and transferred to Immobilon PVDF-FL membrane (Millipore). Membranes were blocked with 5% (w/v) skim milk in Tris-buffered saline with Tween-20 (TBST), pH 8.0, for 30 min and incubated with rabbit pAMPK (1:1,000, Cell Signaling Technology, 2535), mouse AMPK (1:1,000, Cell Signaling Technology, 2793), mouse PP2AC (1:800, Millipore, 05-421), rabbit PP2Cα (1:1,000, Cell Signaling Technology, 3549), rabbit PP1α (1:1,000, Cell Signaling Technology, 2582), rabbit PPM1E (1:500, Santa Cruz Biotechnology, sc-135276), sheep SOD1 (1:4,000, Merck, 574597), rabbit TDP-43 (1:2,000, ProteinTech Group, 10782-2-AP) or mouse β-actin (1:2,000, Sigma) antibodies in 3% (w/v) BSA in TBST overnight at 4°C. Blots were washed three times in TBST for 10 min and incubated with IRDye 680 or 800CW conjugated secondary antibodies (Li-Cor) (1:10,000) followed by three washes in TBST

for 10 min and detected on the Odyssey Classic infrared imaging system. Rabbit IRDye 680-tagged pAMPK and IRDye 800-tagged AMPK antibodies (1:10,000, Dr. John Scott) were used for cell samples and were incubated in 5% (w/v) BSA in TBST overnight at 4°C, washed three times in TBST for 10 min and detected on the Odyssey Classic infrared imaging system. All blots were quantified by taking the mean density of pAMPK bands normalised to total AMPK after subtracting background intensity. PP2AC levels were normalised to β-actin levels. Results were expressed as percentage of WT controls (100%).

Immunocytochemistry

Cells were cultured for 2 days and fixed with 4% paraformaldehyde (PFA) for 10 min, permeabilised in 0.4% TX-100 in phosphate-buffered saline (PBS) for 10 min, blocked in 5% normal goat serum in PBS for 30 min and incubated with rabbit phospho-AMPK (1:100, Cell Signaling Technology) antibody in blocking buffer overnight at 4°C, washed three-times with PBS, for 10 min and incubated with Alexa Fluor 488 conjugated secondary antibody (1:1,000, Molecular Probes) for 2 hr, stained with Hoechst 33342 (1:10,000, Invitrogen) for 15 min, washed three times with PBS for 10 min and mounted using fluorescent mounting medium (Dako) on glass slides (Thermo Scientific) for microscopy using an Olympus FV 1000 confocal microscope. Images were captured using identical exposure and gain settings. Negative controls without primary antibodies were performed which produced no staining.

Immunohistochemistry

Mice were transcardially perfused with PBS followed by 4% (w/v) PFA in 0.1 M phosphate buffer. Lumbar spinal cords were dissected out, post-fixed in 4% (w/v) PFA for 2 hr, processed, dehydrated and embedded in paraffin and cut into 20 μm transverse sections. Sections were deparaffinised, treated with 20 μg/ml Proteinase K (Qiagen) in PBS at 37°C for 5 min for antigen retrieval, permeabilised in 0.4% TX-100 in PBS for 10 min, blocked in 5% normal goat serum in PBS for 30 min and incubated with rabbit phospho-AMPK (1:100, Cell Signaling Technology) and mouse NeuN (1:1,000, Millipore, MAB377) antibodies in blocking buffer overnight at 4°C. Sections were washed three times with PBS for 10 min, incubated with Alexa Fluor 488 and Alexa Fluor 594 conjugated secondary antibodies (1:1,000, Molecular Probes) for 2 h, stained with Hoechst 33342 (1:10,000, Invitrogen) for 15 min, and washed three times with PBS for 10 min before mounting using fluorescent mounting medium onto glass slides for confocal microscopy. The number of cells with pAMPK granules was counted from 30 motor neurons per mouse, 3 mice per group, and expressed as a percentage of WT (100%). The number of pAMPK granules per cell was counted from 30 motor neurons per mouse, 3 mice per group.

Statistical analysis

Western densitometry data were analysed by unpaired t-test or one-way ANOVA with Tukey's post-hoc test. Motor neuron and pAMPK granule quantification was analysed by unpaired t-test. All statistical tests were performed with GraphPad Prism software (version 5.0, GraphPad Software, San Diego, CA).

Results

AMPK activation is increased in spinal cords of symptomatic mutant SOD1 mice

We first determined AMPK activation status in SOD1^{G93A} mice at presymptomatic (P60) and symptomatic (P90) ages. SOD1^{G93A} mice appear normal at P60 and develop hindlimb muscle weakness and wasting at P90. AMPK activation by phosphorylation of α subunit threonine-172 was assessed by immunoblotting for phospho (pAMPK) and total AMPK levels in tissues of SOD1^{G93A} mice and WT littermates. Western blot analysis showed pAMPK and AMPK bands at 62 kDa as expected (Fig. 1). AMPK activation, determined by pAMPK/AMPK ratio, was similar in spinal cords of P60 mice of both genotypes (Fig. 1A,B). However, AMPK activation in spinal cords of SOD1^{G93A} mice was 300% greater than in spinal cords of WT mice at P90 (Fig. 1B, $p<0.05$). There was no change in AMPK activation level in brains of P60 and P90 SOD1^{G93A} mice, however (Fig. 1C,D).

To determine whether increased AMPK activation is selective to spinal cord, we next examined pAMPK/AMPK ratio in non-affected tissues such as liver and kidney. AMPK activation was elevated by 60% and 80% in liver of SOD1^{G93A} mice at P60 and P90, respectively, which was statistically significant at P90 (Fig. 1E,F, $p<0.05$). AMPK activation was also significantly increased by 80% in kidney of SOD1^{G93A} mice at P60 (Fig. 1G,H, $p<0.01$), while unchanged at P90. Thus, AMPK activation occurs at symptom onset in spinal cords of SOD1^{G93A} mice and in peripheral tissues, suggesting that AMPK signalling and therefore energy depletion is a late event in pathogenesis and not restricted to degenerating neurons in this mouse model of ALS.

AMPK activation is reduced in spinal cords and brains of mutant TDP-43 mice

AMPK activation was then examined in TDP-43^{A315T} mice at presymptomatic (P60) and symptomatic (P90) stages. TDP-43^{A315T} mice appear healthy at P60 and develop an abnormal swimming gait at P90 without gross muscle wasting. In contrast to SOD1^{G93A} mice, AMPK activation was drastically decreased by 60% at P60 ($p<0.05$) and 30% at P90 in spinal cords of TDP-43^{A315T} mice (Fig. 2A,B). In brain, pAMPK/AMPK ratios were significantly diminished by 80% at P60 ($p<0.001$) and 60% at P90 ($p<0.05$) in TDP-43^{A315T} mice (Fig. 2E,F). AMPK activation was not studied in liver and kidney, which do not express the TDP-43^{A315T} transgene in our hands and others [32]. Hence, mutant TDP-43 leads to reduced activation of AMPK in spinal cord and brain which was more severe in the latter tissue, consistent with pronounced cortical pathology in these mice [32].

We next screened expression level of phosphatases that could be responsible for dephosphorylation of AMPK in TDP-43^{A315T} mice. We examined four candidate AMPK phosphatases: protein phosphatase 2A (PP2A), PP2C, PP1 and PPM1E. The levels of PP2C, PP1 and PPM1E were similar in spinal cords of presymptomatic and symptomatic TDP-43^{A315T} mice compared to WT (Fig. 2C). However, levels of PP2A were significantly increased by 120% in spinal cords of TDP-43^{A315T} mice at P60 ($p<0.05$), positively correlating with AMPK inactivation (Fig. 2C,D). This suggests that PP2A is the likely phosphatase responsible for AMPK inactivation in TDP-43^{A315T} mice and this phosphatase remained the focus of this study. We also assessed PP2A expression in SOD1^{G93A} mice, showing that PP2A levels were significantly diminished by 30% in spinal cords of mice at P90 ($p<0.05$), showing an inverse correlation with AMPK activation (Fig. 1A,I). This strengthens the evidence that PP2A is modulating AMPK activation in the CNS of mutant SOD1 and TDP-43 mice.

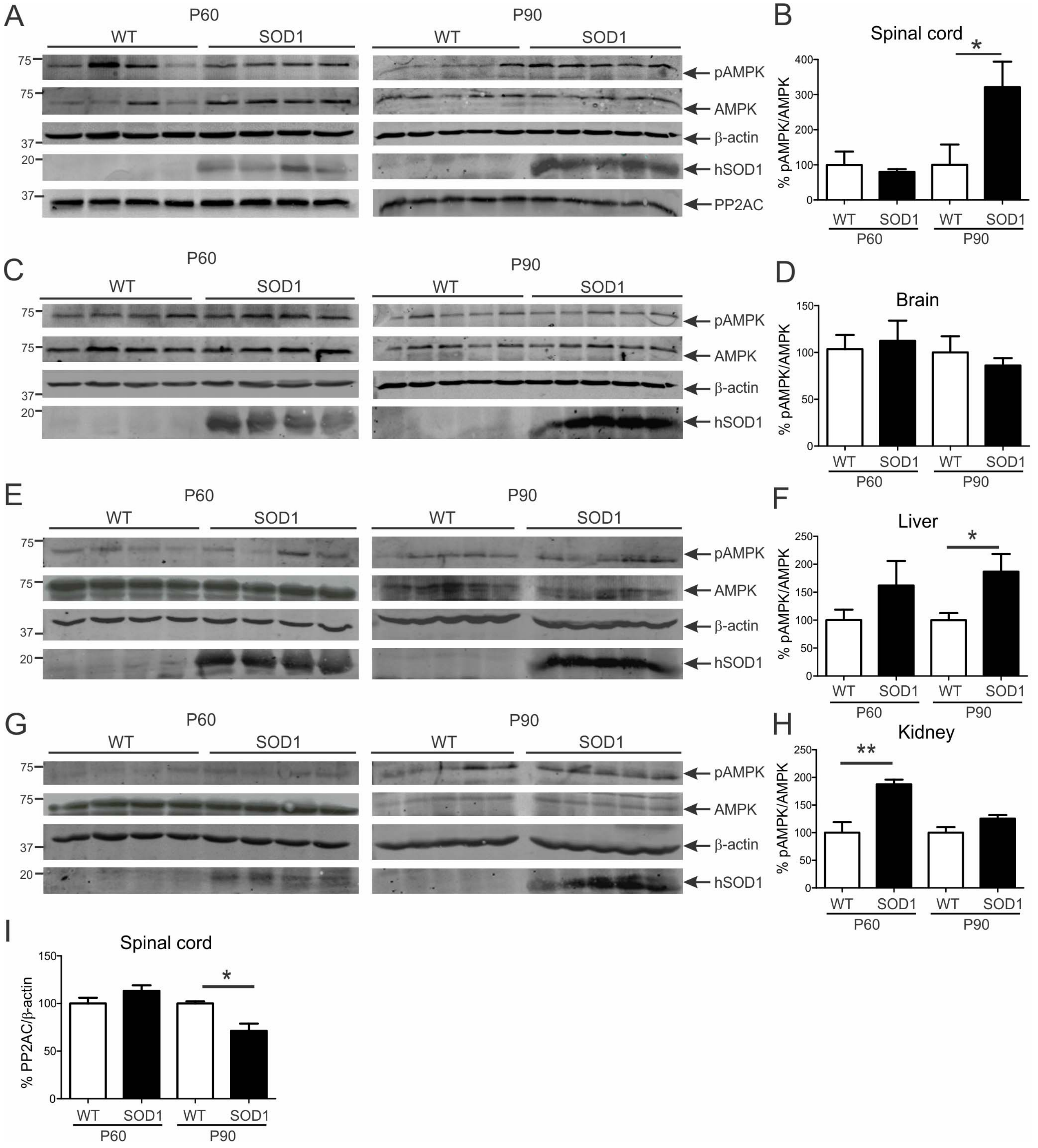

Figure 1. AMPK activation is increased in spinal cords of symptomatic mutant SOD1 mice, but not pre-symptomatic mice. Immunoblot analysis of phosphorylated (pAMPK) and total AMPK levels in **A** spinal cord, **C** brain, **E** liver and **G** kidney of pre-symptomatic (P60) and symptomatic (P90) transgenic SOD1^{G93A} and age-matched wild-type (WT) mice. Quantification of pAMPK/AMPK ratio level in **B** spinal cord, **D** brain, **F** liver and **H** kidney from immunoblots normalised to WT mice. Immunoblot analysis of **A**, PP2A and **I**, quantification of PP2A level from immunoblots for spinal cord. Data represent mean ± SEM, $n = 4$–5 mice per group, $^*p<0.05$ and $^{**}p<0.01$ compared to WT mice using unpaired t-test.
doi:10.1371/journal.pone.0090449.g001

Activated AMPK distribution in spinal motor neurons of normal mice and ALS mouse models

The subcellular distribution of AMPK was examined in spinal cord sections of mice at P90 using immunohistochemistry. In WT mice, there were small pAMPK-positive punctae in the cytoplasm of motor neurons, identified by their size, ventral horn location and NeuN immunoreactivity (Fig. 3, arrowheads). In SOD1^{G93A} mice, there was increased pAMPK immunoreactivity in the

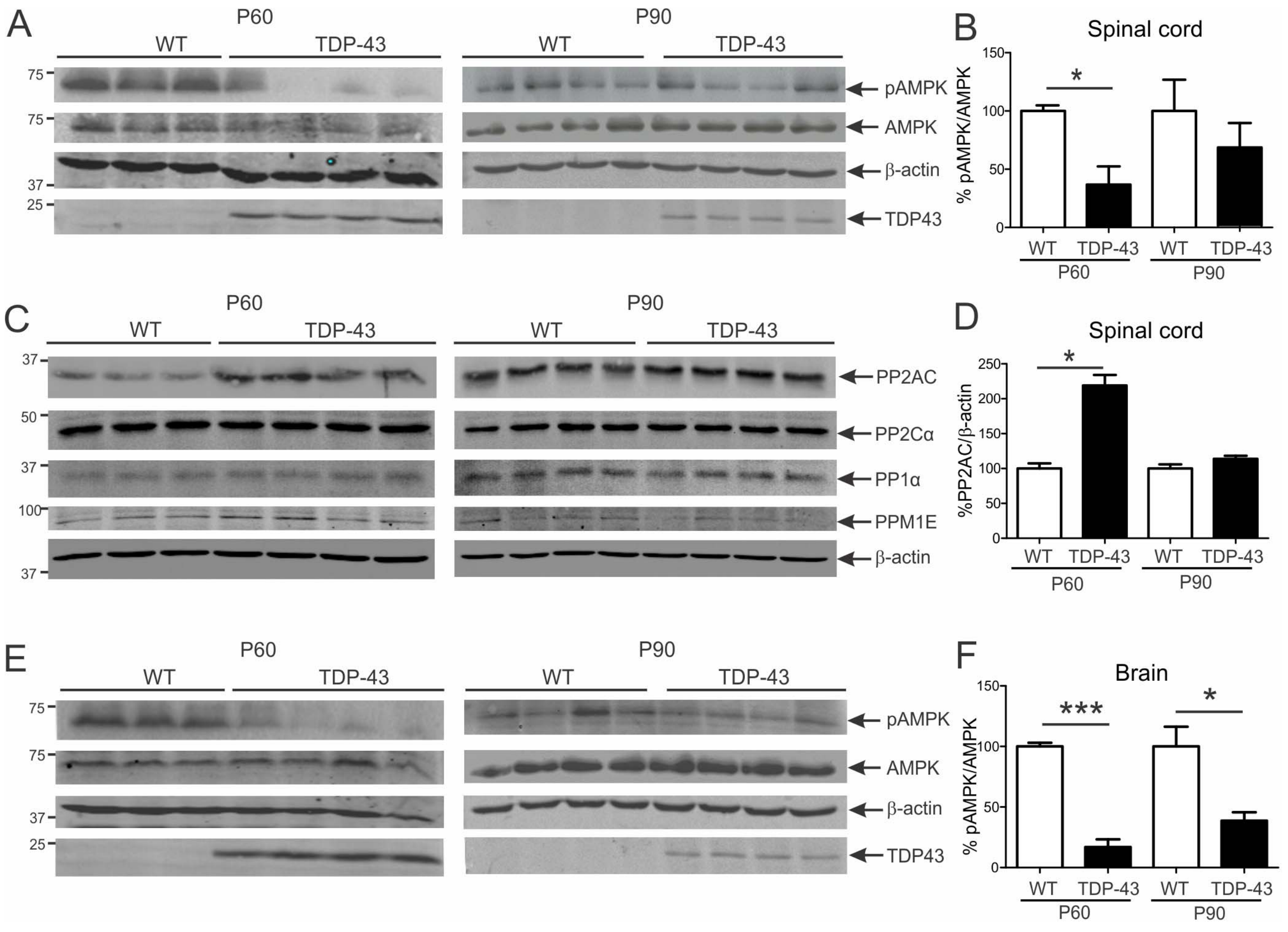

Figure 2. AMPK activation is diminished in spinal cords and brains of mutant TDP-43 mice from pre-symptomatic age. Immunoblot analysis of phosphorylated (pAMPK) and total AMPK levels in **A,** spinal cord and **E,** brain of pre-symptomatic (P60) and symptomatic (P90) transgenic TDP-43^{A315T} and age-matched WT mice. Quantification of pAMPK/AMPK ratio level in **B,** spinal cord and **F,** brain from immunoblots normalised to WT mice. Immunoblot analysis of **C,** PP2A, PP2C, PP1 and PPM1E with **E,** quantification of PP2A level from immunoblots for spinal cord. Data represent mean ± SEM, $n = 4$–5 mice per group, *$p<0.05$ and ***$p<0.001$ compared to WT mice using unpaired t-test.
doi:10.1371/journal.pone.0090449.g002

cytoplasm of motor neurons (Fig. 3A), consistent with Western blotting findings (Fig. 1A,B). Increased pAMPK was restricted to motor neurons and was not observed in NeuN-negative Hoechst-positive cells. We quantified the percentage of motor neurons with cytoplasmic pAMPK granules, showing that almost all motor neurons in WT and SOD1^{G93A} mice contained pAMPK punctae, consistent with the ubiquitous expression of AMPK (Fig. 3B). However, the number of pAMPK granules was doubled in motor neurons of SOD1^{G93A} mice compared to WT mice ($p<0.05$), indicative of increased AMPK activation (Fig. 3C).

In TDP-43^{A315T} mice, pAMPK immunoreactive staining in motor neurons was less than in WT mice (Fig. 3D), again concordant with Western results (Fig. 2A,B). The number of motor neurons containing pAMPK granules was reduced by 50% in TDP-43^{A315T} mice (Fig. 3E, $p<0.05$). Furthermore, there was severe depletion of pAMPK granules by 80% in motor neurons of mutant TDP-43 mice (Fig. 3F, $p<0.05$), consistent with AMPK inactivation in spinal cords of these mice. Thus, activated AMPK is predominantly found in motor neurons and localised to cytoplasmic granules in the spinal cord and abnormally expressed in mutant SOD1 and TDP-43 mouse models of ALS.

AICAR stimulates activation of AMPK in NSC-34 cells

We next studied whether similar changes in AMPK activation occur in motor neuronal NSC-34 cells expressing different ALS-linked SOD1 and TDP-43 mutations. The physiological activation response to the AMPK agonist AICAR was first established in NSC-34 cells. Treatment of NSC-34 cells with AICAR for 2 hr resulted in dose-dependent phosphorylation of AMPK with maximal stimulation at 2 mM concentration (Fig. 4A). Immunocytochemical analysis of untreated NSC-34 cells revealed that pAMPK was primarily and diffusely localised to the cytoplasm (Fig. 4B). There was a dose-dependent increase in cytoplasmic pAMPK immunoreactivity with AICAR treatment. AICAR treatment did not adversely affect NSC-34 cell viability (Fig. 4C). Thus, activated AMPK is mainly cytoplasmic in NSC-34 cells and motor neurons in spinal cord.

AMPK activation in NSC-34 cells expressing SOD1 mutants

AMPK activation in NSC-34 cells stably transfected with dismutase active (G37R, G93A) or inactive (A4V, G85R) human SOD1 mutants was compared to NSC-34 cells expressing WT human SOD1. Although transient transfection with these mutants

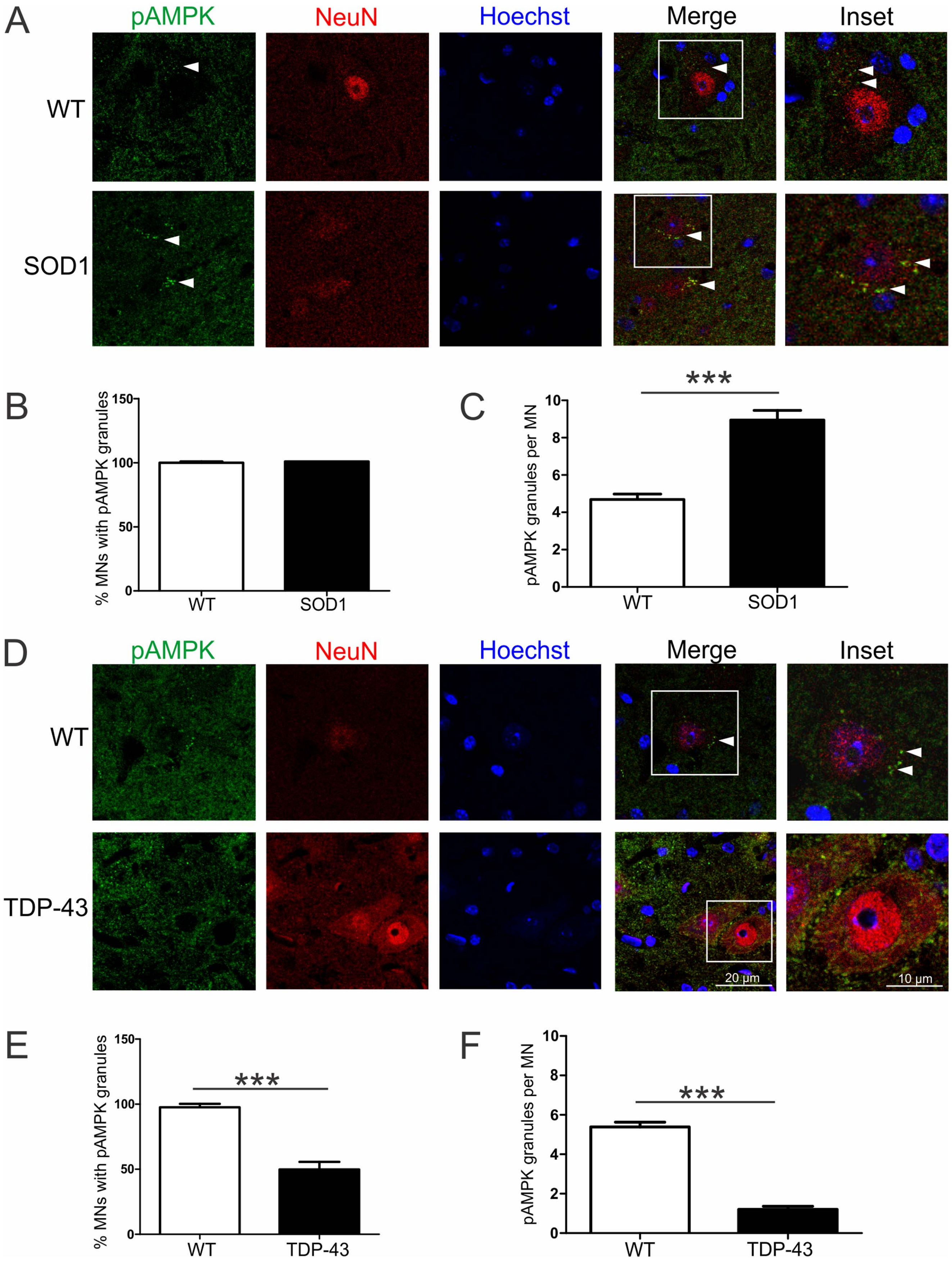
A
pAMPK
NeuN
Hoechst
Merge
Inset
WT
SOD1
B
% MNs with pAMPK granules
150
100
50
0
WT
SOD1
C
***
pAMPK granules per MN
10
8
6
4
2
0
WT
SOD1
D
pAMPK
NeuN
Hoechst
Merge
Inset
WT
TDP-43
20 µm
10 µm
E
% MNs with pAMPK granules
150
100
50
0
***
WT
TDP-43
F
pAMPK granules per MN
10
8
6
4
2
0
***
WT
TDP-43

Figure 3. Activated AMPK distribution in spinal cords of normal, mutant SOD1 and TDP-43 mice. Phosphorylated AMPK (pAMPK) immunohistochemical analysis of spinal cords from P90 mice. **A,** In WT mice, pAMPK is localised to cytoplasmic punctae (arrowhead) in motor neurons identified by NeuN. pAMPK immunoreactive granules accumulate in the cytoplasm (arrowheads) of motor neurons in transgenic SOD1^{G93A} mice. **B**, Quantification of motor neurons (MNs) containing pAMPK granules and **C,** pAMPK granules per motor neuron in WT and SOD1^{G93A} mice. **D**, In transgenic TDP-43^{A315T} mice, cytoplasmic pAMPK immunoreactivity is reduced in motor neurons. **E**, Quantification of motor neurons (MNs) containing pAMPK granules and **F,** pAMPK granules per motor neuron in WT and SOD1^{G93A} mice. Data represent mean ± SEM, $n = 3$ mice per group, $^{***}p<0.001$ compared to WT mice using unpaired t-test.
doi:10.1371/journal.pone.0090449.g003

results in protein aggregates, ER stress and cell death [24,25,26], these features were not found in these stable cells, which may represent a pre-symptomatic ALS state i.e. SOD1^{G93A} mice at P60. In support of this, Western blot analysis showed similar levels of AMPK activation in cells expressing normal or mutant SOD1, although there was a non-significant trend for diminished AMPK

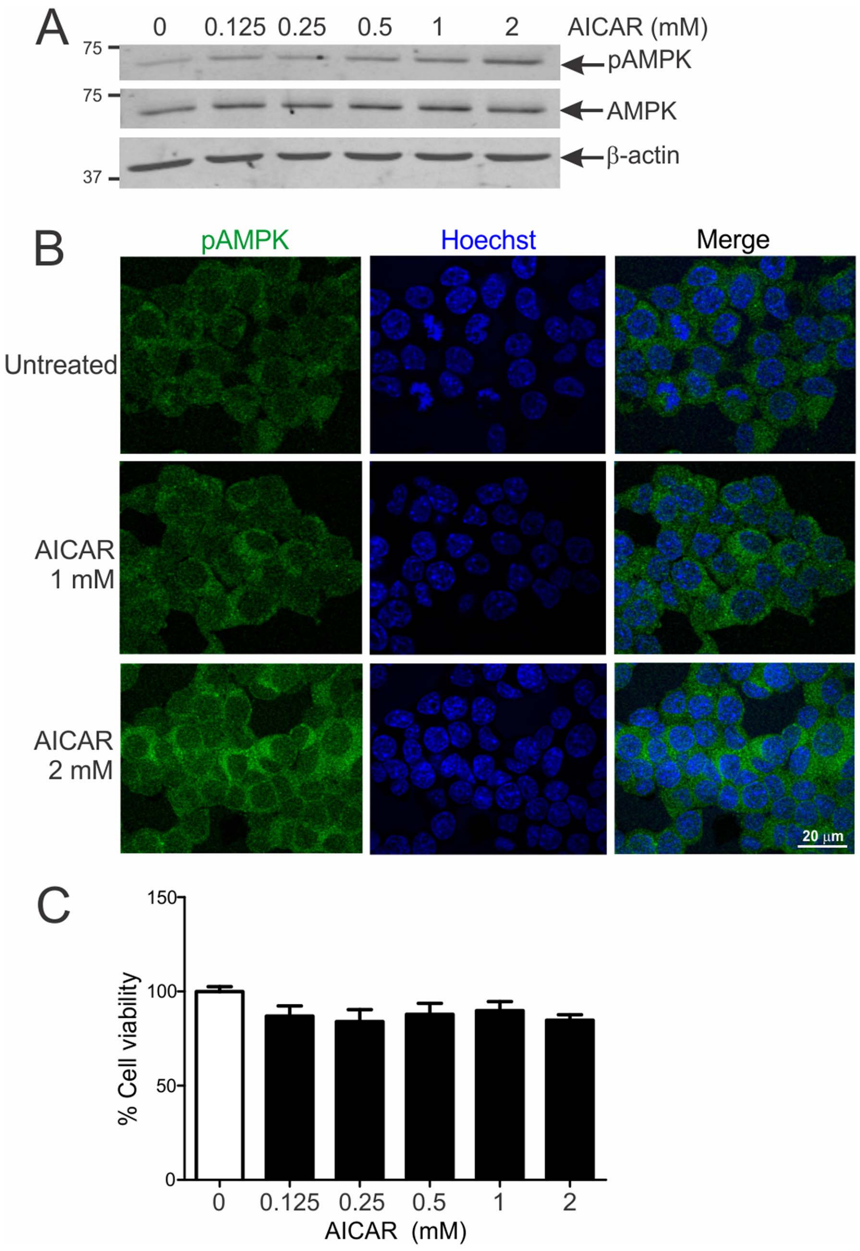

Figure 4. AICAR stimulates activation of AMPK in NSC-34 cells. A, Immunoblot analysis of phosphorylated (pAMPK) and total AMPK levels in cells treated with 0–2 mM AICAR for 2 hr. **B**, Immunocytochemical analysis of untreated cells shows predominantly cytoplasmic pAMPK. Cells treated with AICAR reveal dose-dependent and increased cytoplasmic pAMPK immunoreactivity. **C**, Survival analysis of cells treated with 0–2 mM AICAR for 2 hr. Data represent mean ± SEM, $n = 3$ experiments. None of the AICAR treatment groups were significantly different to untreated controls using one-way ANOVA with Tukey's post-hoc test.
doi:10.1371/journal.pone.0090449.g004

activation (Fig. 5A,B). Likewise, PP2A levels were similar in cells expressing WT or mutant SOD1 (Fig. 5C). These Western blot findings were correlated with immunocytochemical analysis, showing that pAMPK is mainly cytoplasmic in NSC-34 cells expressing normal or mutant SOD1 (Fig. 5D). Therefore, AMPK activation does not occur in presymptomatic neuronal cells or mouse models expressing mutant SOD1.

AMPK activation is reduced by mutant TDP-43 in NSC-34 cells by a PP2A-dependent mechanism

Next, AMPK activation in NSC-34 cells stably expressing WT or mutant (D169G, A315T, Q331K and M337V) TDP-43 was analysed. There was greater cytoplasmic accumulation of mutant than WT TDP-43 in these cells (Fig. 6D). pAMPK/AMPK ratio measured by Western blot was significantly reduced in cells expressing different TDP-43 mutants (Fig. 6A,B). Activated AMPK level was 50% and 70% less in cells expressing Q331K and M337V, respectively, than in cells expressing WT TDP-43 (Fig. 6B, $p<0.05$ and $p<0.01$). There was a corresponding increase

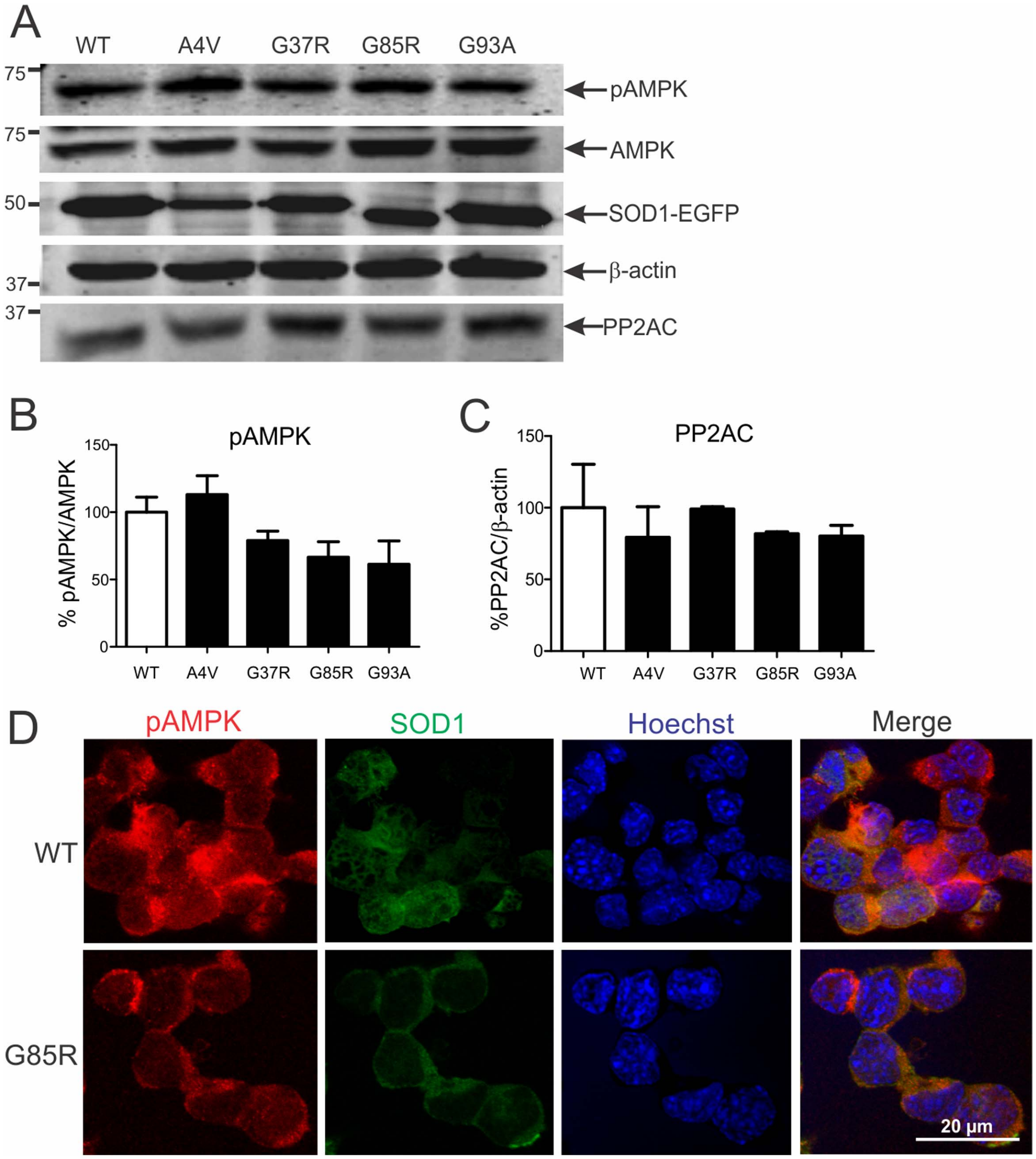

Figure 5. AMPK activation in NSC-34 cells stably expressing normal or mutant SOD1. A, Immunoblot analysis of phosphorylated (pAMPK) and total AMPK in NSC-34 cells stably transfected with human wild-type (WT) or mutant A4V, G37R, G85R or G93A SOD1 reveals similar levels of AMPK activation. **B**, Quantification of pAMPK/AMPK ratio level in cells from immunoblots normalised to WT SOD1 expressing cells. Data represent mean ± SEM, $n = 3$ experiments. **A**, PP2A immunoblot analysis and **C**, quantification of PP2A level from immunoblots. **D**, Immunocytochemical analysis of cells expressing WT or mutant SOD1 shows similar cytoplasmic localisation of pAMPK.
doi:10.1371/journal.pone.0090449.g005

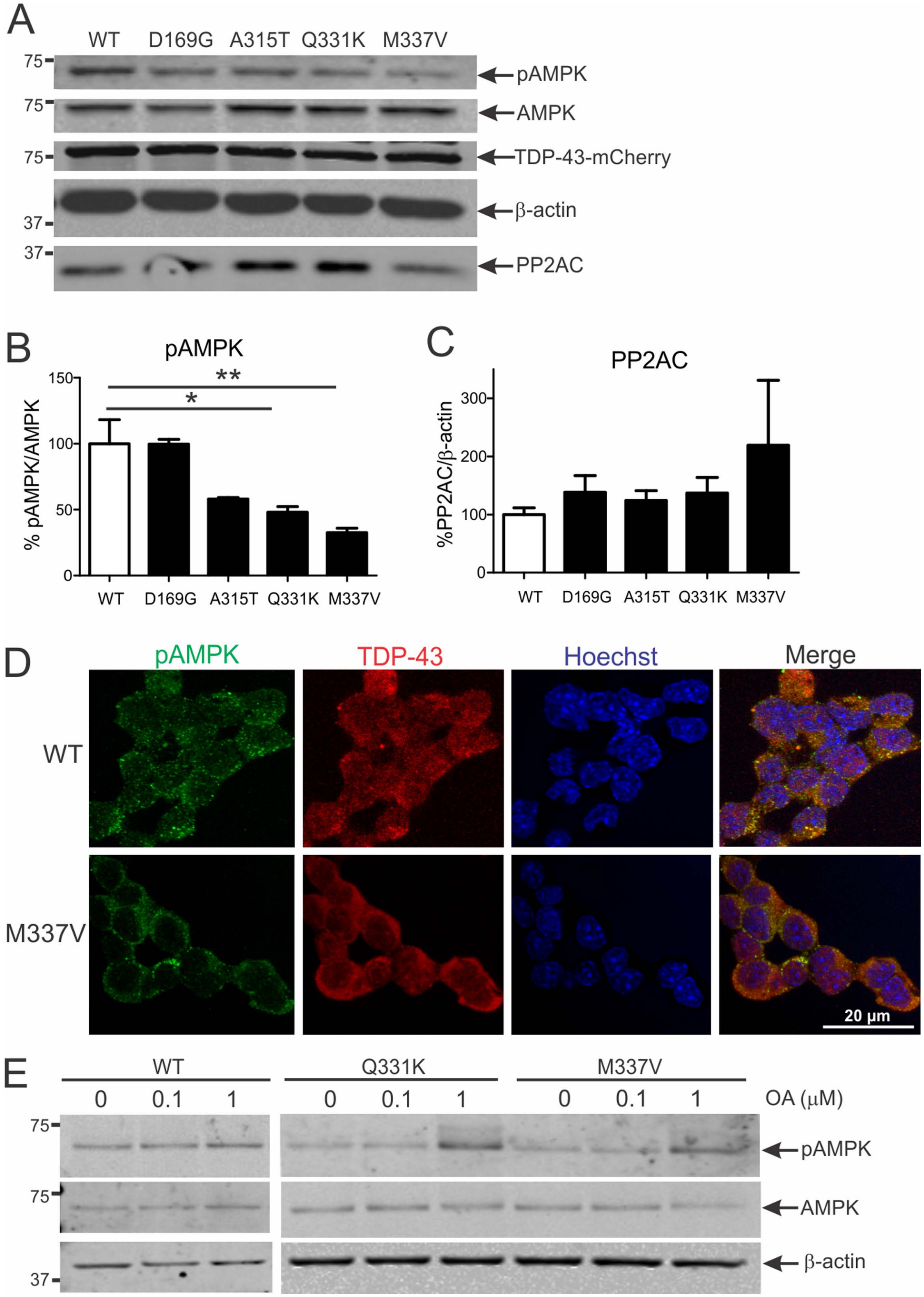

Figure 6. AMPK activation is diminished in NSC-34 cells stably expressing mutant TDP-43 by a PP2A-dependent mechanism. A, Immunoblot analysis of phosphorylated (pAMPK) and total AMPK in NSC-34 cells stably transfected with human wild-type (WT) or mutant D169G, A315T, Q331K and M337V TDP-43. **B**, Quantification of pAMPK/AMPK ratio level from immunoblots normalised to WT TDP-43 expressing cells. Data represent mean ± SEM, $n = 3$ experiments, *$p < 0.05$ and **$p < 0.01$ compared to WT using one-way ANOVA with Tukey's post-hoc test. **A**, PP2A immunoblot analysis and **C**, quantification of PP2A level from immunoblots. **D**, Immunocytochemical analysis of cells expressing WT TDP-43 shows cytoplasmic pAMPK granules which are reduced in mutant TDP-43 expressing cells. **E**, Immunoblot analysis of pAMPK and total AMPK levels in TDP-43 stable cells treated with 0-1 μM okadaic acid (OA) for 30 min.
doi:10.1371/journal.pone.0090449.g006

in the level of PP2A by 40% and 120% in Q331K and M337V expressing cells, respectively (Fig. 6C). In NSC-34 cells expressing WT TDP-43, pAMPK was predominantly located in cytoplasmic granules (Fig. 6D). However, in cells expressing mutant TDP-43, pAMPK immunoreactivity was reduced, consistent with immunoblots and immunohistochemical findings from spinal cords of transgenic TDP-43^{A315T} mice.

To determine whether mutant TDP-43 is driving AMPK inactivation by PP2A, we treated NSC-34 cells expressing WT or mutant TDP-43 with the selective PP2A inhibitor okadaic acid (OA). OA treatment at 1 μM led to increased AMPK activation in cells expressing WT, Q331K or M337V TDP-43, which was more pronounced for mutant TDP-43 (Fig. 6E). Hence, mutant TDP-43 reduction of AMPK activation occurs by a PP2A-dependent mechanism.

Discussion

We demonstrate several novel findings in this study. Firstly, AMPK activation occurs only at disease onset in spinal cords of mutant SOD1 mice, but is not restricted to this tissue, occurring also in non-affected liver and kidney. Secondly, AMPK activation is severely diminished in spinal cords and brains of mice and motor neuronal cells expressing mutant TDP-43. Thirdly, mutant TDP-43 inhibition of AMPK phosphorylation is mediated by PP2A. Lastly, activated AMPK is predominantly localised to small cytoplasmic granules of motor neurons in adult spinal cord.

Elevated AMPK activation in spinal cords of symptomatic SOD1^{G93A} mice correlates well with key indices of energy hypermetabolism reported in this model, including increased glucose and ATP consumption [13], lactate metabolism [2], lipolysis [12] and metabolic acidosis [14]. The presence of activated AMPK in both CNS and peripheral tissues in mutant SOD1 mice at symptom onset argues against a role for AMPK signalling in disease initiation, although it may be a determinant of disease progression. In support of this, treatment of SOD1^{G93A} mice with metformin, which activates AMPK, conferred no significant effect on disease onset, but hastened death in female mice only [27]. Consistent with our findings, increased AMPK activation in spinal cords of P90 mutant SOD1 mice, but not juvenile P40 mice, was recently reported [28]. Here, we resolved the time course of AMPK phosphorylation, showing activation at P90, but not at P60, in these mice. However, this study also found that AMPK-mediated transcription of its downstream target genes (e.g. *PCG-1α*, *GPx1* and *SOD2*) was unaltered in spinal cords of SOD1^{G93A} mice [28] and thus, the role of elevated AMPK signalling in mutant SOD1 mice remains unclear. We suggest that increased AMPK activation in spinal cords of SOD1^{G93A} mice reflects energy depletion in motor neurons well after they start degenerating and is a late event in ALS pathogenesis. This might be brought about by reactivity to muscle wasting and weight loss at symptom onset [29]. Pharmacological activation of AMPK by AICAR or its inhibition by compound C [19] is therefore unlikely to significantly modify disease in mutant SOD1 mice or provide an effective therapeutic benefit in ALS.

In contrast to increased AMPK activation in SOD1^{G93A} mice, its activity was drastically diminished in mice and neuronal cells expressing TDP-43 mutants. While this was an unexpected finding, it can be rationalised in the case of TDP-43^{A315T} mice. Unlike SOD1^{G93A} mice, TDP-43^{A315T} mice develop mild motor neuron loss in the spinal cord and do not progressively lose muscle mass or succumb to ALS-like symptoms, but rather die from bowel obstruction caused by gut innervation defects [30,31]. In contrast, TDP-43^{A315T} mice have pronounced neuronal loss and ubiquitin pathology in cortex, which would be consistent with greater depletion of AMPK activation in brain, compared to spinal cord in this model [32]. Most recently, it was shown that TDP-43^{A315T} mice develop progressive weight gain, increased body fat and adipocyte hypertrophy [18], which is consistent with a state of energy surplus, and therefore AMPK inactivation as shown here. Interestingly, analysis of neuronal cells expressing different TDP-43 mutants also revealed reduced AMPK activation similar to mice. Expression of PP2A, but not other reported AMPK phosphatases, was induced by mutant TDP-43 and treatment with the PP2A inhibitor okadaic acid reversed this effect in cell culture. In another study, PP2A inhibition by okadaic acid or PP2AC knockdown both restored AMPK phosphorylation in cells exposed to heat shock, supporting that AMPK inactivation is driven by PP2A under cell stress [33]. We therefore conclude that PP2A mediates AMPK inactivation observed in cell and mouse models expressing mutant TDP-43. There is increasing evidence that TDP-43 plays an important role in regulating energy metabolism [17,18] and stress-inducible kinases such as c-Jun N-terminal kinase and extracellular-signal-regulated kinase [34] and modulation of PP2A may therefore provide an important link coupling pathogenic TDP-43 to AMPK activity.

In conclusion, our results demonstrate contrasting effects of mutant SOD1 and TDP-43 on AMPK activation in mouse models of ALS. This is likely to reflect key differences in neurodegeneration, resulting muscle wasting and energy metabolism characteristic of these models. In SOD1^{G93A} mice characterised primarily by spinal motor neuron loss, muscle atrophy and weight loss, AMPK activation is increased which is indicative of energy hypermetabolism. In TDP-43^{A315T} mice with mainly cortical motor neuron pathology and minimal spinal motor neuron loss without muscle wasting, AMPK activation is reduced, in keeping with weight gain and fat deposition in this model. Furthermore, we uncover a novel regulation of AMPK activation mediated by PP2A by mutant TDP-43 *in vitro* and *in vivo*. Modulation of PP2A levels in mutant TDP-43 models may therefore present an interesting therapeutic approach in ALS.

Acknowledgments

We thank Dr. Peter Crouch and Assoc. Prof. Anthony White (University of Melbourne) for generously providing TDP-43^{A315T} mice and Dr. Adam Walker and Dr. Julie Atkin (University of Melbourne) for TDP-43-mCherry constructs.

Author Contributions

Conceived and designed the experiments: NDP MKH BJT. Performed the experiments: NDP RKS. Analyzed the data: NDP MKH BJT. Contributed reagents/materials/analysis tools: JWS BEK. Wrote the paper: NDP MKH BJT.

References

1. Ferraiuolo L, Higginbottom A, Heath PR, Barber S, Greenald D, et al. (2011) Dysregulation of astrocyte-motoneuron cross-talk in mutant superoxide dismutase 1-related amyotrophic lateral sclerosis. Brain 134: 2627–41.
2. Ferraiuolo L, Kirby J, Grierson AJ, Sendtner M, Shaw PJ (2011) Molecular pathways of motor neuron injury in amyotrophic lateral sclerosis. Nat Rev Neurol 7: 616–30.
3. Shaw PJ, Eggett CJ (2000) Molecular factors underlying selective vulnerability of motor neurons to neurodegeneration in amyotrophic lateral sclerosis. J Neurol 247: I17–27.
4. Dupuis L, Pradat PF, Ludolph AC, Loeffler JP (2011) Energy metabolism in amyotrophic lateral sclerosis. Lancet Neurol 10: 75–82.

5. Kong J, Xu Z (1998) Massive Mitochondrial Degeneration in Motor Neurons Triggers the Onset of Amyotrophic Lateral Sclerosis in Mice Expressing a Mutant SOD1. J Neurosci 18: 3241–50.
6. Borthwick GM, Johnson MA, Ince PG, Shaw PJ, Turnbull DM (1999) Mitochondrial enzyme activity in amyotrophic lateral sclerosis: Implications for the role of mitochondria in neuronal cell death. Ann Neurol 46: 787–90.
7. Shaw PJ, Eggett CJ (2000) Molecular factors underlying selective vulnerability of motor neurons to neurodegeneration in amyotrophic lateral sclerosis. J Neurol 247: I17–27.
8. Desport JC, Preux PM, Truong TC, Vallat JM, Sautereau D, et al. (1999) Nutritional status is a prognostic factor for survival in ALS patients. Neurology 53: 1059–1063.
9. Vaisman N, Lusaus M, Nefussy B, Niv E, Comaneshter D, et al. (2009) Do patients with amyotrophic lateral sclerosis (ALS) have increased energy needs? J Neurol Sci 279: 26–29.
10. Desport JC, Preux PM, Magy L, Boirie Y, Vallat JM, et al. (2001) Factors correlated with hypermetabolism in patients with amyotrophic lateral sclerosis. Am J Clin Nutr 74: 328–334.
11. Funalot B, Desport JC, Sturtz F, Camu W, Couratier P (2009) High metabolic level in patients with familial amyotrophic lateral sclerosis. Amyotroph Lateral Scler 10: 113–117.
12. Dupuis L, Oudart H, Rene F, Gonzalez de Aguilar JL, Loeffler JP (2004) Evidence for defective energy homeostasis in amyotrophic lateral sclerosis: benefit of a high-energy diet in a transgenic mouse model. Proc Natl Acad Sci U S A 101: 11159–11164.
13. Browne SE, Yang L, DiMauro JP, Fuller SW, Licata SC, et al. (2006) Bioenergetic abnormalities in discrete cerebral motor pathways presage spinal cord pathology in the G93A SOD1 mouse model of ALS. Neurobiol Dis 22: 599–610.
14. Dodge JC, Treleaven CM, Fidler JA, Tamsett TJ, Bao C, et al. (2013) Metabolic signatures of amyotrophic lateral sclerosis reveal insights into disease pathogenesis. Proc Natl Acad Sci U S A 110: 10812–7.
15. Hamadeh MJ, Tarnopolsky MA (2006) Transient caloric restriction in early adulthood hastens disease endpoint in male, but not female, Cu/Zn-SOD mutant G93A mice. Muscle Nerve 34: 709–719.
16. Patel BP, Safdar A, Raha S, Tarnopolsky MA, Hamadeh MJ (2010) Caloric restriction shortens lifespan through an increase in lipid peroxidation, inflammation and apoptosis in the G93A mouse, an animal model of ALS. PLoS One 5: e9386.
17. Chiang PM, Ling J, Jeong YH, Price DL, Aja SM, et al. (2010) Deletion of TDP-43 down-regulates Tbc1d1, a gene linked to obesity, and alters body fat metabolism. Proc Natl Acad Sci U S A 107: 16320–16324.
18. Stallings NR, Puttaparthi K, Dowling KJ, Luther CM, Burns DK, et al. (2013) TDP-43, an ALS linked protein, regulates fate deposition and glucose homeostasis. PLoS One 8: e71793.
19. Li J, McCullough LD (2010) Effects of AMP-activated protein kinase in cerebral ischemia. J Cerebral Blood Flow Metabol 30: 480–492.
20. Ravnskjaer K, Boergesen M, Dalgaard LT, Mandrup S (2006) Glucose-induced repression of PPARalpha gene expression in pancreatic beta-cells involves PP2A activation and AMPK inactivation. J Mol Endocrinol 36: 289–299.
21. Sanders MJ, Grondin PO, Hegarty BD, Snowden MA, Carling D (2007) Investigating the mechanism for AMP activation of the AMP-activated protein kinase cascade. Biochem J 403: 139–48.
22. Garcia-Haro L, Garcia-Gimeno MA, Neumann D, Beullens M, Bollen M, et al. (2010) The PP1-R6 protein phosphatase holoenzyme is involved in the glucose-induced dephosphorylation and inactivation of AMP-activated protein kinase, a key regulator of insulin secretion, in MIN6 beta cells. FASEB J 24: 5090–5091.
23. Voss M, Paterson J, Kelsall IR, Martín-Granados C, Hastie CJ, et al. (2011) Ppm1E is an in cellulo AMP-activated protein kinase phosphatase. Cell Signal 23: 114–24.
24. Turner BJ, Atkin JD, Farg MA, Zang DW, Rembach A, et al. (2005) Impaired extracellular secretion of mutant superoxide dismutase 1 associates with neurotoxicity in familial amyotrophic lateral sclerosis. J Neurosci 25: 108–17.
25. Atkin JD, Farg MA, Turner BJ, Tomas D, Lysaght JA, et al. (2006) Induction of the unfolded protein response in familial amyotrophic lateral sclerosis and association of protein-disulfide isomerase with superoxide dismutase 1. J Biol Chem 281: 30152–65.
26. Soo KY, Atkin JD, Horne MK, Nagley P (2009) Recruitment of mitochondria into apoptotic signaling correlates with the presence of inclusions formed by amyotrophic lateral sclerosis-associated SOD1 mutations. J Neurochem 108: 578–90.
27. Kaneb HM, Sharp PS, Rahmani-Kondori N, Wells DJ (2011) Metformin treatment has no beneficial effect in a dose response survival study in the SOD1G93A mouse model of ALS. PLoS One 6: e24189.
28. Lim MA, Selak MA, Xiang Z, Krainc D, Neve RL, et al. (2012) Reduced activity of AMP-activated protein kinase protects against genetic models of motor neuron disease. J Neurosci 32: 1123–41.
29. Gurney ME, Pu H, Chiu AY, Dal Canto MC, Polchow CY, et al. (1994) Motor neuron degeneration in mice that express a human Cu,Zn superoxide dismutase mutation. Science 264: 1772–1775.
30. Guo Y, Wang Q, Zhang K, An T, Shi P, et al. (2012) HO-1 induction in motor cortex and intestinal dysfunction in TDP-43 A315T transgenic mice. Brain Res 15: 88–95.
31. Esmaeili MA, Panahi M, Yadav S, Hennings L, Kiaei M (2013) Premature death of TDP-43 (A315T) transgenic mice due to gastrointestinal complications prior to development of full neurological symptoms of amyotrophic lateral sclerosis. Int J Exp Pathol 94: 56–64.
32. Wegorzewska I, Bell S, Cairns NJ, Miller TM, Baloh RH (2009) TDP-43 mutant transgenic mice develop features of ALS and frontotemporal lobar degeneration. Proc Natl Acad Sci U S A 106: 18809–14.
33. Wang T, Yu Q, Chen J, Deng B, Qian L, et al. (2010) PP2A mediated AMPK inhibition promotes HSP70 expression in heat shock response. PLoS One 5: e13096.
34. Meyerowitz J, Parker SJ, Vella LJ, Ng DCh, Price KA, et al. (2011) C-Jun N-terminal kinase controls TDP-43 accumulation in stress granules induced by oxidative stress. Mol Neurodegener 6: 57.
